# *Populus balsamifera* Extract and Its Active Component Salicortin Reduce Obesity and Attenuate Insulin Resistance in a Diet-Induced Obese Mouse Model

**DOI:** 10.1155/2013/172537

**Published:** 2013-05-27

**Authors:** Despina Harbilas, Diane Vallerand, Antoine Brault, Ammar Saleem, John T. Arnason, Lina Musallam, Pierre S. Haddad

**Affiliations:** ^1^Canadian Institutes of Health Research (CIHR) Team in Aboriginal Antidiabetic Medicines, Université de Montréal, P.O. Box 6128, Downtown Station, Montréal, QC, Canada H3C 3J7; ^2^Natural Health Products and Metabolic Diseases Laboratory, Department of Pharmacology, Université de Montréal, P.O. Box 6128, Downtown Station, Montréal, QC, Canada H3C 3J7; ^3^Institute of Nutrition and Functional Foods, Université Laval, Quebec City, QC, Canada G1V 0A6; ^4^Montreal Diabetes Research Center, Centre de Recherche du Centre Hospitalier de l'Université de Montréal, Montreal, QC, Canada H1W 4A4; ^5^Department of Biology and Center for Research in Biopharmaceuticals and Biotechnology, University of Ottawa, Ottawa, ON, Canada K1N 6N5

## Abstract

*Populus balsamifera* L. (BP) is a medicinal plant stemming from the traditional pharmacopoeia of the Cree of Eeyou Istchee (CEI—Northern Quebec). *In vitro* screening studies revealed that it strongly inhibited adipogenesis in 3T3-L1 adipocytes, suggesting potential antiobesity activity. Salicortin was identified, through bioassay-guided fractionation, as the active component responsible for BP's activity. The present study aimed to assess the potential of BP and salicortin at reducing obesity and features of the metabolic syndrome, in diet-induced obese C57Bl/6 mice. Mice were subjected to high fat diet (HFD) for sixteen weeks, with BP (125 or 250 mg/kg) or salicortin (12.5 mg/kg) introduced in the HFD for the last eight of the sixteen weeks. BP and salicortin effectively reduced whole body and retroperitoneal fat pad weights, as well as hepatic triglyceride accumulation. Glycemia, insulinemia, leptin, and adiponectin levels were also improved. This was accompanied by a small yet significant reduction in food intake in animals treated with BP. BP and salicortin (slightly) also modulated key components in signaling pathways involved with glucose regulation and lipid oxidation in the liver, muscle, and adipose tissue. These results confirm the validity of the CEI pharmacopoeia as alternative and complementary antiobesity and antidiabetic therapies.

## 1. Introduction

Obesity results from a variety of risk factors, including unhealthy dietary habits and a sedentary lifestyle, resulting in higher energy input than output [1]. It also increases the risk for other chronic illnesses such as type 2 diabetes (T2D) and insulin resistance (IR) [1]. Insulin resistance is characterized by a decreased ability of insulin sensitive tissues to respond to insulin action. Skeletal muscle is the principal tissue involved in glucose metabolism through insulin-dependent or exercise-sensitive glucose transport (Glut4) [2] implicating the Akt [3] and AMPK [4] pathways, respectively. These pathways are also implicated in glucose metabolism in the liver [5–7] and adipose tissue [3, 8–12]. The liver is considered to be the principal tissue involved in glucose storage and production [13]. Adipose tissue synthesizes and stores fatty acids and is recognized as an endocrine organ; releasing adipokines (leptin, adiponectin) that are implicated in glucose and lipid metabolism [14–20]. Obesity not only leads to excessive fat storage in adipose tissue, but also to ectopic fat storage in other insulin sensitive tissues such as the muscle and liver (nonalcoholic fatty liver disease; NAFLD). This, in part, contributes to the development of insulin resistance [21, 22]. 

Several metabolic and signaling pathways are involved in perpetrating the disturbances of obesity and insulin resistance in the three main insulin-sensitive tissues. PPAR*γ* is involved in the differentiation of adipose tissue; inducing lipid accumulation [23]. Other pathways are involved in lipid entry (FAT/CD36, FABP4) [24–27], lipid metabolism (SREBP-1c and FAS) [28, 29], and oxidation (ACC, CPT-1, PPAR*α*, UCP pathways) [30]. The ERK pathway, involved in cell proliferation and differentiation, also seems to play an important role in both the liver (leads to NAFLD) [31] and adipose tissue [32, 33]. The IKK*αβ* pathway is involved in the inflammatory response characteristic of obesity and indirectly mediates insulin resistance [27]. 

In Canada, the Cree of Eeyou Istchee (CEI) of Eastern James Bay have a prevalence of obesity and T2D that is, respectively, at least 1.5 [34, 35] and 4 times higher [36] than the general Canadian population. This may be the consequence of major lifestyle changes (decreased physical activity and gradual adoption of nontraditional diets), as well as cultural difficulty to comply with modern T2D treatments. Our team has been working with the CEI to identify plants stemming from their traditional pharmacopoeia that could offer culturally adapted complementary and alternative treatments for obesity and T2D. As part of an ethnobotanical survey, *Populus balsamifera* L. (Salicaceae) (balsam poplar) was identified as a plant used by the CEI to treat a variety of symptoms associated with T2D. As part of an *in vitro* bioassay platform used to screen for the antidiabetic potential of CEI plants, the 3T3-L1 cell line was selected to assess glitazone-like activity and stimulation of adipogenesis. *Populus balsamifera* L., also known as balsam poplar, unexpectedly and potently inhibited the accumulation of intracellular triglycerides [37–39], suggesting potential antiobesity activity. This plant extract contains a number of active components, namely, salicin, salicortin, salireposide, and populoside [37]. In subsequent studies conducted in the same cell line, a bioassay-guided fractionation approach identified salicortin, a salicylate glycoside, as the principal active component of *P. balsamifera* responsible for the observed inhibition of adipogenesis [39]. Salicortin is abundant in poplar, willow bark, and throughout the Salicaceae family [39]. Although salicylates are well known for having anti-inflammatory properties, improving insulin sensitivity [40–42], and even having antiproliferative effects [43], antiadipogenic activity had never been ascribed prior to the studies conducted by our team. We thus introduced *P. balsamifera* extract, alongside a high-fat diet (HFD), to study the plant's ability to mitigate the development of obesity using the* in vivo* diet-induced obese (DIO) C57BL/6 mouse model. The results clearly demonstrated that the plant extract substantially attenuated weight gain and the development of insulin resistance [44]. 

In the present studies, we sought to evaluate the effectiveness of *P. balsamifera* as well as its active principle salicortin at treating obesity and insulin resistance once they have been established in the same model [45, 46]. As previously described by other researchers [47] and discussed further below, DIO mice respond in a stratified manner to the HFD; some animals being resistant to the HFD (low responders—LR) while others show the clearcut profile of metabolic disease (high responders—HR). *P. balsamifera* and salicortin were thus administered to the latter DIO mice in order to determine their potential effectiveness in countering obesity and insulin resistance. 

## 2. Materials and Methods

### 2.1. Plant Extracts

Specimens of *Populus balsamifera* L. (Salicaceae) were collected on CEI territory (Eastern James Bay, Quebec, Canada). Dr. Alain Cuerrier, taxonomist at the Montreal Botanical Garden, confirmed that the botanical identity and voucher specimens were deposited in the Marie-Victorin herbarium of the Montreal Botanical Garden in Montreal, Canada (Mis03-49). A crude 80% ethanolic extract of *P. balsamifera* was prepared as previously described [37]. Salicortin, the active principle of *P*. *balsamifera*, was produced through fractionation, isolation, and purification of the crude plant extract as previously described [39]. The structure of the purified compound was identified and confirmed by ^1^H and ^13^C NMR and by comparison with previously reported data. 1D- and 2D-NMR spectra were generated using an Avance 400 spectrometer (Bruker Biospin Corporation) [39].

### 2.2. Animals and Diets

Four-week-old male nondiabetic C57BL/6 mice (Charles River Laboratories, Saint-Constant, QC, Canada) were housed in individual cages, maintained on a 12 h light-dark cycle in a temperature and humidity-controlled animal room, and given free access to food and water. Following acclimatization, the mice were divided into groups of approximately 12 mice each. Chow controls received a standard diet (SD; 18% protein content, 4.5% crude fat; Charles River Animal rodent diet) for 16 weeks. Other groups were fed a high fat diet (HFD; Bio-Serv Diet #F3282; 60% energy from fat) for eight weeks. *P. balsamifera* at 125 or 250 mg/kg, and salicortin at 12.5 mg/kg were incorporated in the HFD and treatments continued for an additional 8 weeks (DIO controls receiving only HFD). Balsam poplar extract studies were initiated first while the active compound was being identified, isolated, or purified. Since, salicortin composes 10% of the whole plant extract, and that the most efficient dose of balsam poplar was 125 mg/kg, salicortin was administered at 12.5 mg/kg. Hence, experimental groups of plant extract and salicortin are compared to distinct CHOW nonobese and DIO controls. However, both experimental protocols were conducted in an identical manner, and DIO controls reacted in a fully comparable fashion relative to nonobese Chow congeners in both studies. Body weight, food and water intake, as well as glycemia were measured 3 times/week during the entire study. Glycemia was measured by pricking the tail vein and by using a commercial glucometer (Accu-Check Roche, Montreal, QC, Canada). Measurements were always performed at the same time/day, in the same order and by the same person. All experimental protocols were approved by the animal experimentation ethics committee of the Université de Montréal and were carried out in full respect of the guidelines from the Canadian Council for the Care and Protection of Animals. 

### 2.3. Data and Animal Segregation

The area under the curve (AUC) was calculated for parameters measured in a continuous manner throughout the study. The total AUC was then separated into two parts: fraction 1 (F1), representing AUC between week 0 and 4 (first month of treatment), and fraction 2 (F2) corresponding to the AUC between week 4 and 8 of plant extract administration (second month of treatment). This segregation served to determine the plant extract's temporal course of action, that is, whether early in onset (first 4 weeks), later (last 4 weeks), or present throughout the study. Once the experimental feeding protocol had been carried out, we became aware of the studies of Peyot and collaborators [47] discriminating low responders (LR) and high responders (HR) in the DIO mouse model. As discussed by these authors and observed in our own studies, pooling animals with different characteristics, such as low weight gain, weak IR and near-normal glycemia, with animals with high weight gain, frank IR, and hyperglycemia, can yield misleading results [47]. Therefore, we segregated the DIO animals based on these published criteria and analyzed our data accordingly. As expected, low responder animals exhibited a near normal metabolic profile and treatment with the plant extract or its active component essentially had little if any effect. This is positive in the sense that *P. balsamifera* extract and salicortin may have a desirable safety margin by being active only in metabolically compromised animals. This also confirms the validity of the segregation. Hence, data are presented for the effects of plant extract and active principle in HR animals only. This segregation did however reduce our sample size, hence contributing to data variability.

### 2.4. Surgical Procedure

At the end of the experimental protocol, mice were anesthetized with an intraperitoneal injection of 50 mg/kg pentobarbital and sacrificed by exsanguination. Livers were flushed with physiological saline, dissected, immediately placed in liquid nitrogen, and stored until further use at −80°C. Soleus skeletal muscle, white adipose tissue (WAT; epididymal and retroperitoneal fat pads), subscapular brown adipose tissue (BAT), and kidneys were also collected, placed in liquid nitrogen, and stored at −80°C until further use. 

### 2.5. Blood Parameters

Plasma insulin, adiponectin, and leptin were assessed by radioimmunoassay (RIA: Linco Research, St.Charles, MO, USA). To avoid interrupting dietary plant treatment and disturbing the HFD feeding pattern (hence affecting the DIO model), mice were not fasting when blood parameters were measured. Plasma levels of AST, ALT, LDH, creatinine, alkaline phosphatase, and circulating lipids (triglycerides, total cholesterol, LDL-cholesterol and HDL-cholesterol) were measured by standard clinical biochemistry assays at the Department of Biochemistry of Sainte-Justine's Children Hospital (Montreal, QC, Canada). 

### 2.6. Tissue Triglyceride Measurement

Part of the frozen liver and muscle sections (around 100 mg of each sample) were ground into powder under liquid nitrogen and extracted using Folch's chloroform/methanol (2 : 1) method [48]. Triglyceride content was quantified using a commercial kit (Randox Laboratories Ltd., UK). 

### 2.7. Western Blot Analysis

Western blot analysis was performed on frozen liver, muscle, and WAT using the following antibodies: p-Akt (Ser 473), Akt, p-AMPK (Thr 172), AMPK, Glut4, p-ACC, ACC, FAS, FABP4, phospho p44/42 MAPK, p44/42 MAPK, p-IKK*αβ*, and *β*-actin (each at 1 : 1000 in blocking buffer incubated overnight at 4°C; Cell Signaling Tech Inc., Danvers, MA, USA). PPAR*α*, PPAR*γ*, CPT-1, CD36, UCP-2, and SREBP1-c were measured using a 1 : 200 dilution in blocking buffer and incubated either at 1 h room temperature (RT) or overnight (Santa Cruz Biotechnology Inc., Santa Cruz, CA, USA). The following HRP-conjugated secondary antibodies were used: anti-rabbit (1 : 10000; Jackson Immunoresearch Laboratories Inc., West Grove, PA, USA), anti-mouse (1 : 4000; Cell Signaling Tech Inc., Danvers, MA, USA), or anti-goat (1 : 5000; Santa Cruz Biotechnology inc., Santa Cruz, CA, USA). Immunoreactive proteins were detected by enhanced chemiluminescence method (GE Healthcare, Baie d'Urfé, QC, Canada). Densitometric analysis was performed using NIH Image J software (version 1.42q, NIH, USA).

### 2.8. Statistical Analysis

Data were analyzed by one-way analysis of variance (ANOVA) with Bonferroni post hoc analysis, or by unpaired Student's *t* test (Sigma Stat software, Jandel Scientific, San Rafael, CA, USA), as appropriate. Areas under the curve (AUC) were calculated with PRISM software (GraphPad, San Diego, CA, USA). Data are expressed as the mean ± SEM of the indicated number of determinations. Statistical significance was set at *P* < 0.05. 

## 3. Results

### 3.1. Metabolic Profile of Responders to the High Fat Diet (DIO Controls)

As anticipated in relation to recent data by Peyot et al. [47], roughly half of the mice consuming the HFD became obese and insulin resistant. Compared to Chow controls, such DIO control animals gained body weight, increased liver, BAT and WAT weights (Table 1, *P* < 0.05), and displayed hyperlipidemia (total cholesterol, LDL, HDL). These mice also exhibited increased plasma glucose and insulin levels, an enhanced leptin/adiponectin ratio (Table 2, *P* < 0.05) as well as elevated hepatic and muscle triglyceride (TG) levels (Table 3; *P* < 0.05 compared to Chow), thus confirming the presence of an insulin resistant state. Only mice displaying an altered metabolic profile at 8 weeks of HFD feeding were selected for the present study and randomized to receive *P. balsamifera* or its active component, salicortin, for an additional 8 weeks.

### 3.2. *P. balsamifera* and Salicortin Decrease Body Weight, Liver Weight, and Steatosis in DIO Mice

**Figure 1:**
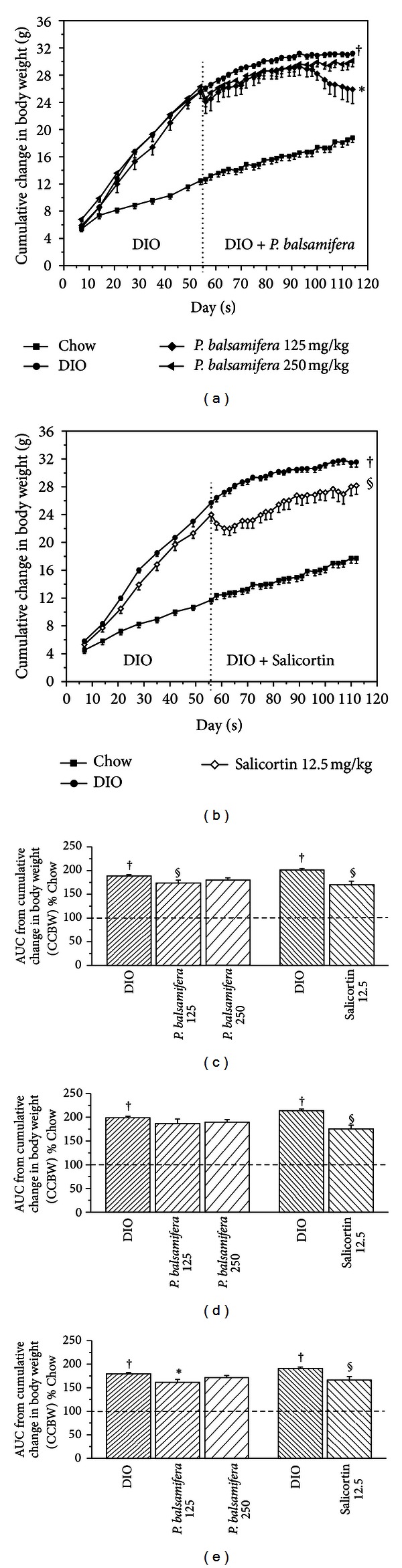
Cumulative changes in body weight (CCBW) in C57BL/6 mice treated with either standard diet (Chow), HFD (DIO), and (a) HFD in combination with *P. balsamifera* at 125 or 250 mg/kg, or (b) HFD in combination with salicortin. Area under the curve (AUC) of CCBW for (c) total 8-week treatment period, (d) first 4 weeks of treatment (F1), and (e) second 4 weeks of treatment (F2). As mentioned, C57BL/6 mice were administered either standard diet (Chow), HFD (DIO), and for the last 8 of the 16 weeks a HFD in combination with *P. balsamifera* at 125 or 250 mg/kg, or with Salicortin 12.5 mg/kg. All values are mean ± SEM. Fraction 1 (F1) consists in the AUC between week 0 and 4, and fraction 2 (F2) corresponds to the AUC between week 4 and 8 of administration of the plant extract. The number of animals for the crude plant extract protocol was CHOW (*n* = 12), DIO (*n* = 8), *P. balsamifera* 125 (*n* = 5), and *P. balsamifera* 250 (*n* = 7); and for the salicortin protocol: CHOW (*n* = 12), DIO (*n* = 7), salicortin (*n* = 9). ^†^denotes DIO significantly different as compared to Chow (unpaired Student's *t* test; *P* < 0.05). *denotes significantly different as compared to respective DIO (one way ANOVA; *P* < 0.05). ^§^denotes significantly different as compared to respective DIO (unpaired Student's *t* test; *P* < 0.05).

Treatment with *P. balsamifera* (at 125 mg/kg) significantly decreased body weight. This decrease reached 13% at sacrifice when compared to DIO controls (*P* < 0.05; Table 1). When taking into account continuous measurements of cumulative changes in body weight (CCBW; Figures 1(a) and 1(b)), the area under the curve (AUC) was lowered by 8% (*P* < 0.05; Figure 1(c)) with 125 mg/kg of *P. balsamifera*. This effect was gradual, beginning within the first month (*F*1 = 6% reduction; N.S.; Figure 1(d)), but becoming more pronounced in the second month of the treatment (*F*2 = 10% decrease; *P* < 0.05; Figure 1(e)). Animals receiving 250 mg/kg of *P. balsamifera* exhibited a similar pattern of effects, albeit without reaching statistical significance. Mice fed the active compound, salicortin, also displayed a significant decrease in body weight amounting to an 8% drop at sacrifice (*P* < 0.05; Table 1) and a 15% decrease in total AUC of CCBW (*P* < 0.05; Figure 1(c)). Interestingly, its effect was immediate with 18% decrease in the F1 AUC (*P* < 0.05; Figure 1(d)) that continued into the second month, albeit slightly less prominently (*F*2 = 13%; *P* < 0.05; Figure 1(e)). 

Concomitantly, epididymal fat pad weight increased, whereas retroperitoneal fat pad weight was smaller when animals were treated with either dose of balsam poplar (9%–17% reduction) or with salicortin (10% decrease) than in those receiving HFD alone, although the latter changes failed to reach statistical significance (Table 1; N.S). In contrast, the drop in liver weight was significant in the three aforementioned treated groups (decrease by 27%–35%; *P* < 0.05 versus DIO controls; Table 1). Consistent with these results, hepatic triglyceride (TG) content was also reduced by 44% to 53% in the treated animals in comparison to DIO controls (*P* < 0.05; Table 3). Muscle triglycerides, however, were not significantly altered by any of the treatments (N.S.; Table 3). 

It must be noted that a weak anorexic effect was observed in animals receiving *P. balsamifera* at 125 mg/Kg dose. Indeed, the AUC of cumulative food intake of this group was significantly reduced by 6% as compared to DIO congeners (*P* < 0.05, data not shown). No such effect was observed with the higher dose of *P. balsamifera* or with salicortin. 

### 3.3. *P. balsamifera* and Salicortin Improve Insulin Sensitivity, While only the Active Principle Modulates Lipidemia in DIO Mice

**Figure 2:**
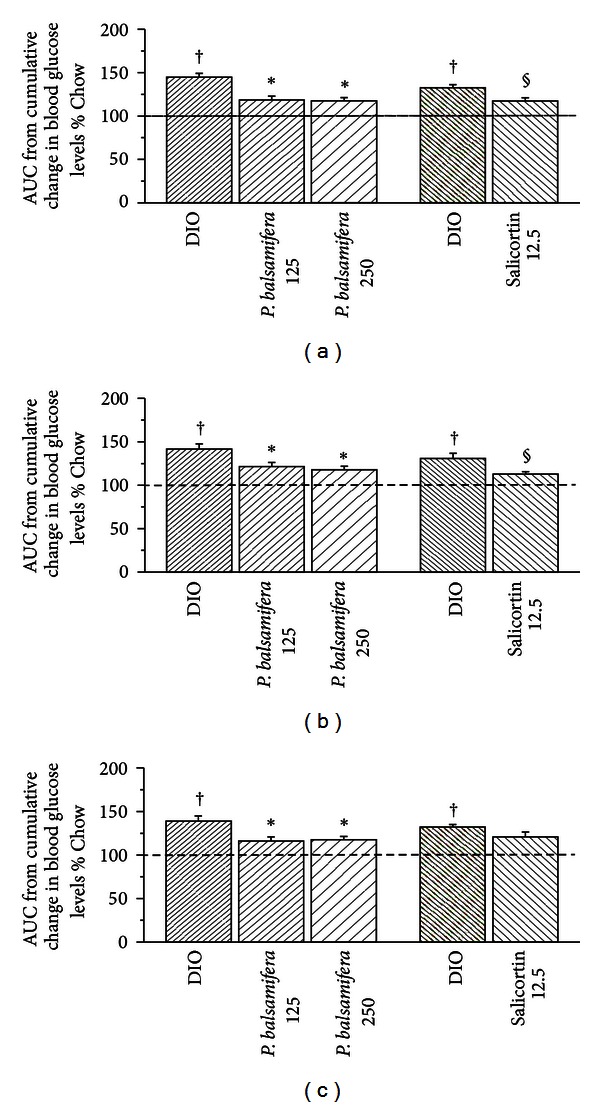
Area under the curve (AUC) of cumulative changes in blood glucose levels for (a) total 8-week treatment period, (b) first 4 weeks of treatment (F1), and (c) second 4 weeks of treatment (F2). C57BL/6 mice were administered either standard diet (Chow), HFD (DIO), and for the last 8 of the 16 weeks a HFD in combination with *P. balsamifera* at 125 or 250 mg/kg, or with salicortin 12.5 mg/kg. All values are mean ± SEM. Fraction 1 (F1) consists in the AUC between week 0 and 4, and fraction 2 (F2) corresponds to the AUC between week 4 and 8 of administration of the plant extract. The number of animals for the crude plant extract protocol was CHOW (*n* = 12), DIO (*n* = 8), *P. balsamifera* 125 (*n* = 5), and *P. balsamifera* 250 (*n* = 7); and for the salicortin protocol: CHOW (*n* = 12), DIO (*n* = 7), salicortin (*n* = 9). ^†^denotes DIO significantly different as compared to Chow (unpaired Student's *t* test; *P* < 0.05). *denotes significantly different as compared to respective DIO (one way ANOVA; *P* < 0.05). ^§^denotes significantly different as compared to respective DIO (unpaired Student's *t* test; *P* < 0.05).

Along with body weight changes,* P. balsamifera* (at 125 and 250 mg/kg) and salicortin improved insulin sensitivity, albeit with slightly different profiles. Firstly, continuous measurement of glycemia showed that both doses of whole plant extract as well as the active principle significantly reduced total AUC by 17%-18% and by 11%, respectively. Although *P. balsamifera* and salicortin effects were rather rapid in onset, the effect of the whole plant was constant throughout the treatment period (*F*1 = *F*2), whereas that of the active principle decreased with time (*F*1 = 14%, *P* < 0.05 versus DIO controls; *F*2 = 9%, N.S.; Figures 2(b) and 2(c)). At sacrifice, glycemia of the three treatment groups was reduced as compared to their respective controls, albeit not in a statistically significant manner (Table 2). Secondly, insulinemia diminished by 85% with balsam poplar at 125 mg/Kg (*P* < 0.05) and by 73% with 250 mg/Kg (*P* = 0.052) as well as with salicortin (*P* < 0.05) in comparison to DIO controls (Table 2). Thirdly, the two doses of *P. balsamifera* decreased leptin/adiponectin ratio by 41%–54% as compared to congeners receiving HFD alone (*P* < 0.05; Table 2). Salicortin also significantly decreased this ratio, although to a lesser extent (by 21%; *P* < 0.05; Table 2). In terms of the circulating lipid profile, only the salicortin treated group exhibited significantly lowered total plasma cholesterol and LDL levels, which were reduced by 25% and 40%, respectively (Table 2; *P* < 0.05) as compared to the DIO controls. Altogether, these findings illustrate an improvement in insulin sensitivity when balsam poplar or its active principle are added to the HFD.

Finally, *P. balsamifera* and salicortin tended to normalize several systemic parameters of toxicity, although this did not reach statistical significance, except in the case of AST levels for salicortin (*P* < 0.05; Table 2) and LDH levels for *P. balsamifera* at 125 mg/Kg (*P* < 0.05; Table 2). 

### 3.4. *P. balsamifera* Tends to Increase Skeletal Muscle Glut4 and Improves Components Related to Muscle Lipid Oxidation without Affecting the Akt and AMPK Pathways, Whereas Salicortin Tends to Increase Akt Phosphorylation and Activates p44/42 MAPK

Despite the significant reduction of overall glycemia exerted by the plant extract and its active principle, analysis of protein components involved in muscle glucose homeostasis did not exhibit any statistically significant changes. There was a tendency for Glut4 expression to increase in animals treated with *P. balsamifera* at 125 mg/kg, (Table 4; N.S. balsam poplar versus corresponding DIO controls). Similarly, insulin-dependent Akt phosphorylation tended to increase in animals fed with salicortin, although data variability precluded any definitive interpretation of these results. The insulin-independent AMPK pathway remained more clearly unchanged. 

In contrast, components involved in muscle lipid homeostasis showed evidence of improvement with balsam poplar treatment. Indeed, *P. balsamifera* at 125 mg/kg more than doubled muscle PPAR*α* expression levels (Table 4; 137% increase compared to DIO *P* < 0.05). When looking at components involved in muscle fatty acid oxidation and synthesis, again only the plant extract seemed to act on such pathways, by tending to increase phosphorylated ACC levels and to normalize FAS levels back down to Chow levels (Table 4; N.S. compared to DIO controls). The p44/42 MAPK pathway linked to exercise and insulin stimulation was significantly activated with salicortin (Table 4, *P* < 0.05), and showed a tendency to do so with the plant extract at 250 mg/kg (Table 4, N.S.).

### 3.5. The Effects of *P. balsamifera* and Salicortin on Liver Components of Glucose and Lipid Homeostasis

Both doses of *P. balsamifera* significantly increased hepatic phosphorylated Akt in HFD-fed animals (Table 4; increases by 111% and 87% for 125 and 250 mg/kg groups, respectively; *P* < 0.05 compared to DIO controls), while the active principle showed only a slight tendency to do so (22% increase). A number of parameters related to hepatic lipid homeostasis or inflammation showed interesting tendencies, but none of these effects reached statistical significance. In all cases, tendencies were more pronounced with the 125 than the 250 mg/kg dose of *P. balsamifera*. Notably, PPAR*α* appeared to be increased by both balsam poplar and the active principle, while CPT-1 seemed to be increased only by the plant extract (Table 4, N.S.). As for IKK*αβ* it appeared to be affected only by the plant extract, exhibiting a decrease of 43% and 30% with 125 and 250 mg/kg doses, respectively (Table 4; N.S.). 

### 3.6. The Effect of *P. balsamifera* and Salicortin on Adipose Tissue Components of Glucose and Lipid Homeostasis

*P. balsamifera* at 125 mg/kg showed a strong tendency to increase phosphorylated Akt levels in adipose tissue (Table 4; increase by 65%, *P* = 0.068 compared to DIO controls). Likewise, CPT-1 expression in animals treated with the plant extract at 250 mg/kg exhibited a strong tendency to be enhanced (Table 4; increase by 47%, *P* = 0.079 compared to DIO), whereas the active principle had a similar albeit much weaker effect on this parameter (Table 4; 11% increase; N.S.). In contrast, FABP4 was clearly and significantly increased by *P. balsamifera* at both doses (54% and 60% at 125 and 250 mg/kg, respectively, Table 4, *P* < 0.05 compared to DIO controls), while salicortin showed only a slight tendency to do so (16%, N.S., Table 4;). Salicortin and balsam poplar showed a tendency to normalize PPAR*γ* and phosphorylated p44/42 MAPK to levels similar to those observed in Chow animals (Table 4). Other components failed to show any significant changes in plant or active-principle treated treated animals compared to their respective DIO controls. 

## 4. Discussion

According to the World Health Organization (WHO), 75% of the world population still relies on traditional medicine for primary health care needs and this often involves crude preparations of medicinal plants [49]. In the Canadian province of Quebec, regional health authorities assigned to the CEI are currently considering the usefulness of Cree traditional medicine, notably its associated pharmacopoeia, to deal with several health concerns such as type 2 diabetes; a condition that has reached epidemic proportions in the region [36]. Our group has been working since 2003 with communities and health authorities in CEI to build the scientific evidence base in support of this initiative. An ethnobotanical study was conducted in collaboration with CEI Elders and healers that identified several plants used to treat diabetes symptoms [37, 50, 51]. One of these was *Populus balsamifera* L. (Salicaceae) or balsam poplar. The plant did not demonstrate much antidiabetic potential in *in vitro* bioassays; for instance, it had little effect on muscle glucose uptake [37]. However, the plant caught our attention by its complete inhibition of triglyceride accumulation and adipogenesis in the 3T3-L1 adipocyte cell line [37], suggesting potential therapeutic usefulness against obesity. Salicortin, a salicylate glycoside, abundant in poplar, willow bark, as well as throughout the Salicaceae family, was identified through bioassay-guided fractionation as the constituent of *P. balsamifera* having the most potential to inhibit adipogenesis in the 3T3-L1 cell line [39]. Prior to our studies, antiadipogenic activity had never been ascribed to balsam poplar, to members of its botanical family, or to its known phytochemical constituents, such as salicortin [39]. The goal of the present study was to evaluate the effectiveness of balsam poplar and salicortin as antiobesity, antiadipogenic, and consequently antidiabetic agents in an *in vivo* mouse model.

 The DIO mouse model was used in this study. It closely mimics human metabolic syndrome (notably obesity and insulin resistance) and requires lesser quantities of plant extracts (also, more importantly, of active principles) than larger animals for long-term studies. Indeed, in this model, a period of 8 weeks is necessary to establish obesity and insulin resistance, as confirmed in the present studies. The plant was then incorporated into the high fat diet for a further 8 weeks to fully assess its potential to treat obesity and the associated metabolic disturbances. After 16 weeks on a high fat diet, control DIO animals develop obesity, mild hyperglycemia, hyperinsulinemia, hyperleptinemia, and increased ectopic fat storage (notably hepatic steatosis), all reflecting the establishment of the metabolic syndrome and an insulin resistant state. In previous studies, a less severe model was used whereby animals were subjected to a HFD for only 8 weeks; *P. balsamifera* being administered from the onset of the HFD feeding in order to evaluate its potential to prevent obesity and its associated insulin resistant state [44]. The plant extract effectively reduced body weight gain, retroperitoneal fat pad weight, liver lipid content, as well as circulating glucose, insulin and leptin levels. It also activated pathways that were involved with glucose and lipid oxidation, as well as thermoregulation. The onset of action of the plant extract was immediate and sustained throughout its course of administration. 

The results of the current study clearly demonstrate that in mice subjected to a continuous hypercaloric fat-laden diet, *P. balsamifera* significantly reduced body weight, whereas its active salicortin prevented further weight gain. The plant's effect was more potent and statistically significant at 125 mg/kg than 250 mg/kg. Several anthropomorphic, systemic and tissue parameters were thus examined to circumscribe the possible mechanisms of action of the plant extract and its active principle, salicortin. 

A first potentially important lead came from data on cumulative food intake. Indeed, the plant extract at 125 mg/kg slightly but significantly reduced energy intake, and this was visible in the second month of treatment (F2; data not shown). This correlated well with the plant's temporal action on body weight. An initial reduction in body weight was observed upon introduction of the plant extract in the diet and may have resulted from a behavioral response to the food change. However, body weight rapidly resumed its course such that cumulative weight gain in the first month period (F1) was not significantly different among treatment groups. This contrasts with the reduced energy intake in the second month period (F2) that coincided with a significant decrease in weight gain. Such results suggested that the plant may exhibit slight appetite-modifying effects. Interestingly, these putative anorexic effects were seen only with the 125 mg/kg dose and indicate an unconventional dose-response relationship, as discussed further below. Nevertheless, such anorexic effects warrant further investigation. Notably, appetite-related hormones, such as leptin, as well as gut-brain appetite control mechanisms will need to be examined. 

However, the reduction in caloric intake was weaker than the weight loss measured, roughly half to two-thirds as important when considering total or F2 AUC measurements of cumulative weight changes, respectively. In contrast, the active salicortin decreased the overall AUC of CCBW without affecting cumulative energy intake. This not only suggests different profiles of biological activity between the active principle and the plant extract, but also that other phytochemical components present within balsam poplar are contributing to its appetite-modifying effect.

On the other hand, although obesity was only partly countered by *P. balsamifera* and salicortin, systemic glucose homeostasis was more significantly improved. Indeed, continuous glycemia measurements showed that the plant and its active principle had an overall effect to reduce blood glucose variations toward normal values observed in Chow-fed controls. Even more telling was the dramatic decrease of insulinemia seen with *P. balsamifera* at 125 mg/kg and with salicortin. Likewise, the leptin-to-adiponectin ratio, also reflective of insulin resistance, was essentially halved with the plant extract and decreased by 1.5-fold with the active principle. Interestingly, salicortin also significantly improved the blood lipid profile by decreasing LDL and total cholesterol levels, whereas the plant extract had no significant impact on systemic parameters of lipid homeostasis. This again points to variations in biological activity between the crude extract and the purified active principle.

Further analysis of the major insulin responsive tissues, notably skeletal muscle, liver and adipose tissue, yielded data that highlights potential mechanisms at several levels of metabolic control. Firstly, excessive skeletal muscle TG accumulation was not corrected by *P. balsamifera* or salicortin treatment. In fact, if anything, balsam poplar extract at 125 mg/kg tended to increase this parameter, albeit large variations in the data preclude any definite interpretations. One possibility is that the two-month treatment was not sufficient to significantly affect muscle TG accumulation, yet improvements in muscle lipid and glucose metabolism could have been initiated. Indeed, the crude plant extract did more than double the expression of PPAR*α*, which could lead to increased fatty acid oxidation [30] and improved muscle insulin sensitivity [52]. In animals receiving 125 mg/kg of the plant, muscle Glut4 expression tended to increase and this is consistent with enhanced insulin sensitivity. In contrast, salicortin treatment only significantly affected muscle p44/42 MAPK activation, again hinting at different actions of the plant extract and its active principle. 

In contrast, in the liver, *P. balsamifera* and salicortin treatment more than halved the elevated levels of accumulated TGs. Since hepatic steatosis is increasingly recognized as a major contributor to systemic insulin resistance [53, 54], this action may have played a significant role in improving systemic glucose homeostasis and insulin sensitivity. Indeed, analysis of key tissue proteins indicated that *P. balsamifera* treatment induced a doubling of liver Akt phosphorylation. Since Akt is a major component of the insulin-signaling cascade, part of the effect of balsam poplar could involve improved hepatic insulin sensitivity. Indeed, Akt inhibits glucose production and promotes glycogen deposition in the liver [5, 6, 13]. In hepatic cell lines, our group recently found that *P. balsamifera* inhibits glucose-6-phosphatase [55]. Other components also tended to be modulated by *P. balsamifera* in the liver and suggested that the plant may favor salvaging lipid metabolism. Indeed, PPAR*α* levels were increased by treatment with the plant and its active principle, this transcription factor being known to enhance fatty acid oxidation [30]. The tendency for a reduction of IKK*αβ* by the plant treatment, on the other hand, points to a potential improvement of inflammatory components known to be involved in nonalcoholic fatty liver disease and ensuing metabolic disturbances [27, 56]. Such effects of *P. balsamifera* and salicortin on liver lipid homeostasis and inflammation will require confirmation in future studies. 

Despite large reductions in retroperitoneal fat pad weight at sacrifice, consistent with the significant reduction in body weight, such changes induced by *P. balsamifera* and salicortin failed to reach statistical significance due to data variability. In contrast, epididymal fat pad weight was reduced in DIO mice relative to Chow controls and this was normalized by balsam poplar extract treatment. The paradoxical decrease in epididymal fat pad weight in DIO mice may reflect the redistribution of fat towards more visceral sites in response to the high fat diet as observed by other investigators [57, 58]. 

On the other hand, obesity, especially visceral, leads to low-grade inflammation, releasing into circulation proinflammatory cytokines that contribute to the development of insulin resistance and diabetes. In addition, since both balsam poplar and salicortin belong to the salicylates family, well known for their anti-inflammatory properties, it would be of interest to evaluate the effect of these treatments on circulating proinflammatory cytokines (e.g., TNF-*α*, IL-1*β*, IL-6, resistin, C-reactive protein, and so on). Our group recently assessed the effects of *P. balsamifera* on TNF-*α* production in THP-1 monocytes (ATCC TIB-202). Preliminary results indicate that *P. balsamifera* displays moderate anti-inflammatory properties in LPS-stimulated THP-1 monocytes [59]. 

Nonetheless, analysis of adipose tissue components yielded a number of insightful results. Firstly, the tendency for *P. balsamifera* and salicortin to reduce the p44/42 ERK MAP kinase is consistent with the parallel tendency for WAT weight reductions. Indeed, the ERK pathway is involved in adipogenesis and insulin resistance [32, 60] and our group observed that *P. balsamifera* inhibits clonal expansion in 3T3-L1 adipocytes [38]. On the other hand, FABP-4, a lipid chaperone carrying fatty acids to cellular pathways of oxidation, was significantly increased by plant treatment. As in liver, adipose tissue Akt and CPT-1 expression also showed a strong tendency to be increased, supporting the notion that *P. balsamifera* can enhance insulin-dependent lipid oxidative pathways. The active principle showed much weaker actions on adipose tissue components, notably mild tendencies to increase FABP-4 and CPT-1 expression. 

The effects of both the crude plant extract and of the active principle salicortin occurred without any overt sign of toxicity, albeit balsam poplar extract tended to increase blood creatinine whereas salicortin tended to reduce the same parameter. Future studies should assess kidney function in a more detailed manner. However, unaltered liver function parameters support the interpretation that the plant and its active salicortin are fairly innocuous. Indeed, products of this tree have been used safely for generations by several Aboriginal peoples of the Northern hemisphere [61, 62]. The inner bark of *P. balsamifera* (from which the plant extract used in the current studies was derived) is even documented as a survival food [62, 63]. 

Interestingly, the majority of metabolically and statistically significant changes were obtained with the lower dose of 125 mg/kg of *P. balsamifera*, whereas the larger 250 mg/kg dose exerted lesser or no effects. Such counterintuitive dose-response relationships are not uncommon with polymolecular drug mixtures. Synergistic and antagonistic interactions may occur between the phytochemical components, yielding unconventional dose-response profiles [64]; for instance, observing an anorexic effect at the 125 mg/kg dose but not at that of 250 mg/kg. Such interactions are also supported by the aforementioned differences in the biological activity profile between the crude plant extract and salicortin. It is conceivable that other components in the crude extract may complement salicortin's activity.

Indeed, the action of the active principle alone on continuously measured parameters (body weight and blood glucose) appeared to wane with time, since effects were more pronounced in the first month of administration (F1) than in the second (F2). This may limit the use of the active principle at this dose and may have contributed to mask effects on protein components in insulin-sensitive tissues. Further studies need to be conducted in order to determine if this apparent time-dependent decline in activity develops at any dosage, and if so, with what time course. Nevertheless, salicortin has a sufficiently promising biological profile in DIO mice to warrant further studies potentially leading to clinical assessments.

In summary, *P. balsamifera* and salicortin exerted significant weight-reducing properties in obese, insulin resistant mice in the face of continued HFD feeding. Part of the plant extract's effect appears to emanate from a putative weak anorexic effect that will need to be defined, taking into consideration the loss of effect with higher doses. The plant and the active principle had even more profound beneficial effects on systemic glucose homeostasis and indirect indices of insulin sensitivity. Analysis of tissue components involved in glucose and lipid homeostasis uncovered several potential lead mechanisms in key insulin responsive organs such as skeletal muscle, liver, and adipose tissue. Generally, components involved in insulin-dependent lipid oxidative pathways were most prominently and coordinately modulated in animals treated with the plant extract. Such actions would favor the “wastage” of energy derived from excess lipids consumed through the HFD, thereby reducing the negative metabolic impact of obesity. This is highly relevant for Aboriginal populations like the CEI whose rapid changes in dietary habits over the last decades also involve a higher intake of lipid-enriched calorie-dense foods. It is noteworthy that salicortin did not always activate the same pathways and to the same degree as the plant extract, suggesting that other plant constituents in the crude extract may also participate in beneficial biological activity toward metabolic disease. 

In conclusion, the present studies confirm the high potential of *P. balsamifera* as a complementary treatment derived from CEI traditional medicine, which can help combat the devastating effects of obesity, often leading to type 2 diabetes. Having identified salicortin as an important active principle *in vitro*—its anti-obesity and mild antidiabetic effects having also been validated by the present *in vivo* treatment study—it can now be considered as a valuable tool to ensure the quality and efficacy of *P. balsamifera* preparations. Salicortin can also serve as a template to develop novel therapeutic agents for the treatment of obesity and type 2 diabetes. Additional studies should further clarify the mode of action of the plant and its active principle. This will pave the way toward clinical studies designed to determine if *P. balsamifera* and salicortin can be used in a safe and efficacious manner, alongside conventional medical treatments, for the treatment of metabolic diseases. 

## Figures and Tables

**Table 1:** Effects of obesity, *P. balsamifera*, and salicortin treatments on body and organ weights at sacrifice.

	DIO	*P. balsamifera *125 mg/kg	*P. balsamifera * 250 mg/kg	DIO	Salicortin 12.5 mg/kg
Body Weight	138 ± 1^†^	120 ± 7*	131 ± 3	142 ± 2^†^	130 ± 3^§^
Retroperitoneal Fat Pad	229 ± 12^†^	190 ± 28	209 ± 20	242 ± 13^†^	218 ± 10
Epididymal Fat Pad	77 ± 3^†^	98 ± 17	103 ± 5*	97 ± 2	149 ± 11^§^
Brown Adipose tissue	189 ± 14^†^	136 ± 22 *P* = 0.057	168 ± 14	200 ± 6^†^	170 ± 11^§^
Liver Weight	167 ± 6^†^	108 ± 12*	122 ± 10*	166 ± 9^†^	118 ± 7^§^
Total Kidney	111 ± 4^†^	93 ± 3*	104 ± 4	104 ± 2	101 ± 3

Measurements were obtained after 16 weeks of treatment with either standard diet (Chow), HFD (DIO), and for the last 8 of the 16 weeks with HFD in combination with *P. balsamifera* at 125 or 250 mg/kg, or with the active salicortin at 12.5 mg/kg. All values are expressed as a percentage of respective Chow controls (reference set at 100%) and represent the mean ± SEM. The number of animals for each group for the *P. balsamifera* protocol was: CHOW (*n* = 12); DIO (*n* = 8); *P. balsamifera* 125 (*n* = 5); *P. balsamifera* 250 (*n* = 7); and for the salicortin protocol: CHOW (*n* = 12); DIO (*n* = 7); salicortin (*n* = 9). ^†^denotes DIO significantly different as compared to Chow (unpaired Student's *t* test; *P* < 0.05). *denotes significantly different as compared to respective DIO (one way ANOVA, Bonferroni *post hoc* test; *P* < 0.05). ^§^denotes significantly different as compared to respective DIO (unpaired Student's *t* test; *P* < 0.05).

**Table 2:** Effects of obesity, *P. balsamifera*, and salicortin treatments on systemic parameters at sacrifice.

	DIO	*P. balsamifera * 125 mg/kg	*P. balsamifera *250 mg/kg	DIO	Salicortin 12.5 mg/kg
Glucose (mmol/L)	135 ± 14^†^	105 ± 8	121 ± 10	121 ± 6^†^	114 ± 6
Insulin (ng/mL)	3056 ± 1074^†^	450 ± 238*	832 ± 423 (*P* = 0.052)	1035 ± 150^†^	272 ± 62^§^
Leptin (ng/mL)	211 ± 28^†^	108 ± 18*	145 ± 9 (*P* = 0.051)	246 ± 19^†^	197 ± 13^§^
Adiponectin (*μ*g/mL)	70 ± 3^†^	78 ± 10	82 ± 5 (*P* = 0.054)	97 ± 5	101 ± 6
Leptin/adiponectin ratio	304 ± 37^†^	138 ± 16*	181 ± 16*	248 ± 12^†^	196 ± 18^§^
TG (mmol/L)	99 ± 8	80 ± 10	82 ± 8	118 ± 10	106 ± 14
LDL (mmol/L)	391 ± 31^†^	355 ± 37	307 ± 42	344 ± 20^†^	207 ± 22^§^
HDL (mmol/L)	141 ± 8^†^	112 ± 17	125 ± 8	157 ± 10^†^	137 ± 7
Total cholesterol (mmol/L)	180 ± 9^†^	152 ± 14	151 ± 12	203 ± 11^†^	151 ± 7^§^
ALT (U/L)	281 ± 39^†^	229 ± 47	271 ± 77	341 ± 123^†^	157 ± 27
AST (U/L)	172 ± 30^†^	153 ± 22	137 ± 22	163 ± 27^†^	86 ± 5^§^
Creatinine (U/L)	184 ± 50	491 ± 160	557 ± 138	276 ± 113	136 ± 30
Alkaline phosphatase (U/L)	115 ± 20	85 ± 15	100 ± 9	106 ± 9	88 ± 17
LDH (U/L)	341 ± 83^†^	137 ± 16^§^	193 ± 61	376 ± 156^†^	154 ± 38

Measurements were obtained after 16 weeks of treatment with either standard diet (Chow), HFD (DIO), and for the last 8 of the 16 weeks with HFD in combination with *P. balsamifera* at 125 or 250 mg/kg, or with the active salicortin at 12.5 mg/kg. All values are expressed as a percentage of their respective Chow controls (reference set at 100%) and represent the mean ± SEM. The number of animals for each group for the *P. balsamifera* protocol was: CHOW (*n* = 12); DIO (*n* = 8); *P. balsamifera* 125 (*n* = 5); *P. balsamifera* 250 (*n* = 7); and for the salicortin protocol: CHOW (*n* = 12); DIO (*n* = 7); salicortin (*n* = 9). ^†^denotes DIO significantly different as compared to Chow (unpaired Student's *t* test; *P* < 0.05). *denotes significantly different as compared to respective DIO (one way ANOVA, Bonferroni *post hoc* test; *P* < 0.05). ^§^denotes significantly different as compared to respective DIO (unpaired Student's *t* test; *P* < 0.05).

**Table 3:** Effects of obesity, *P. balsamifera*, and salicortin treatments on hepatic and muscular triglyceride accumulation.

	DIO	*P. balsamifera* 125 mg/kg	*P. balsamifera* 250 mg/kg	DIO	Salicortin 12.5 mg/kg
Liver TG Levels (mg/g total liver)	930 ± 65^†^	436 ± 146*	521 ± 116*	1084 ± 180^†^	559 ± 93^§^
Muscle TG levels (*μ*g/mg)	223 ± 54^†^	342 ± 81	267 ± 38	230 ± 32^†^	219 ± 24

The colorimetric dosage of TG levels in both the liver and muscle was determined using a commercial kit (Randox Laboratories ltd). Measurements were obtained after 16 weeks of treatment with either standard diet (Chow), HFD (DIO), and for the last 8 of the 16 weeks with HFD in combination with *P. balsamifera* at 125 or 250 mg/kg, or with the active salicortin at 12.5 mg/kg. All values are expressed as percentage of respective Chow (reference set at 100%) and represent the mean ± SEM. The number of animals for each group for the *P. balsamifera* protocol was: CHOW (*n* = 12); DIO (*n* = 8); *P. balsamifera* 125 (*n* = 5); *P. balsamifera* 250 (*n* = 7); and for the salicortin protocol: CHOW (*n* = 12); DIO (*n* = 7); salicortin (*n* = 9). ^†^denotes DIO significantly different as compared to Chow (unpaired Student's *t* test; *P* < 0.05). *denotes significantly different as compared to respective DIO (one way ANOVA, Bonferroni *post hoc* test; *P* < 0.05). ^§^denotes significantly different as compared to respective DIO (unpaired Student's *t* test; *P* < 0.05).

**Table 4:** Effects of obesity, *P. balsamifera, * and salicortin treatments on tissue components involved in glucose and lipid homeostasis.

	DIO	*P. balsamifera* 125 mg/kg	*P. balsamifera* 250 mg/kg	DIO	Salicortin 12.5 mg/kg
Muscle					
Glut4	150 ± 63	321 ± 174	151 ± 52	70 ± 33	59 ± 7
pAkt/Akt	214 ± 59^†^	195 ± 47	267 ± 82	96 ± 23	120 ± 29
phospho p44/42 Mapk/44/42Mapk	178 ± 83	99 ± 15	273 ± 84	47 ± 11	146 ± 34^§ ^
pAMPk/AMPk	138 ± 43	79 ± 10	97 ± 22	108 ± 16	108 ± 22
pACC/ACC	122 ± 28	171 ± 56	157 ± 38	106 ± 22	89 ± 26
PPAR*α*/*β*-actine	97 ± 23	229 ± 50^§^	143 ± 44	195 ± 90	162 ± 69
FAS/*β*-actine	118 ± 30	100 ± 39	77 ± 18	114 ± 16	143 ± 29

Liver					
pAkt/Akt	66 ± 14	139 ± 18*	124 ± 17*	106 ± 28	129 ± 37
phospho p44/42 Mapk/44/42Mapk	68 ± 21	48 ± 21	66 ± 14	109 ± 35	96 ± 44
pACC/ACC	105 ± 41	117 ± 74	114 ± 62	71 ± 26	77 ± 31
PPAR*α*/*β*-actine	63 ± 6^†^	88 ± 20	75 ± 13	107 ± 26	147 ± 26
UCP-2/*β*-actine	94 ± 19	107 ± 29	88 ± 26	151 ± 32	149 ± 27
CPT-l/*β*-actine	84 ± 12	100 ± 8	86 ± 9	86 ± 6	83 ± 9
FAS/*β*-actine	69 ± 17	69 ± 22	59 ± 25	86 ± 27	74 ± 15
SREBPl-c/*β*-actine	88 ± 16	112 ± 27	121 ± 37	109 ± 14	109 ± 17
CD36/*β*-actine	63 ± 7^†^	93 ± 19	64 ± 3	69 ± 8^†^	67 ± 6
pIKK*αβ*/*β*-actine	108 ± 23	62 ± 31	75 ± 12	114 ± 21	129 ± 21

Adipose tissue					
pAkt/Akt	112 ± 13	186 ± 37 (*P* = 0.068)	110 ± 15	138 ± 18	109 ± 14
phospho p44/42 Mapk/44/42Mapk	133 ± 31	103 ± 12	79 ± 10	156 ± 23^†^	128 ± 21
PPAR*γ*/*β*-actine	73 ± 17	74 ± 15	102 ± 30	85 ± 12	116 ± 15
pACC/ACC	139 ± 46	119 ± 66	157 ± 70	95 ± 30	86 ± 20
CPT-l/*β*-actine	81 ± 9	86 ± 10	119 ± 19 (*P* = 0.079)	89 ± 11	99 ± 14
FABP4/*β*-actine	85 ± 11	131 ± 11^§^	136 ± 19^§^	71 ± 6	83 ± 10
FAS/*β*-actine	40 ± 5^†^	33 ± 8	56 ± 11	49 ± 7^†^	45 ± 7
SREBP-1 c/*β*-actine	77 ± 6	88 ± 9	96 ± 19	86 ± 7	87 ± 11

Samples of muscle, liver, and WAT were obtained after 16 weeks of treatment with either standard diet (Chow), HFD (DIO), and for the last 8 of the 16 weeks with HFD in combination with *P. balsamifera* at 125 or 250 mg/kg, or with the active salicortin at 12.5 mg/kg. The samples were homogenized and analyzed by immunoblotting. Blots were quantified by densitometry. All values are expressed as percentage of respective Chow (reference set at 100%) and represent the mean ± SEM. The number of animals for each group for the *P. balsamifera* protocol was: CHOW (*n* = 12); DIO (*n* = 8); *P. balsamifera* 125 (*n* = 5); *P. balsamifera* 250 (*n* = 7); and for the salicortin protocol: CHOW (*n* = 12); DIO (*n* = 7); salicortin (*n* = 9). ^†^denotes DIO significantly different as compared to Chow (unpaired Student's *t* test; *P* < 0.05). *denotes significantly different as compared to respective DIO (one way ANOVA, Bonferroni *post hoc* test; *P* < 0.05).^§^denotes significantly different as compared to respective DIO (unpaired Student's *t* test; *P* < 0.05).
